# A bibliometric and visual analysis of research trends and hotspots of familial hypertrophic cardiomyopathy: A review

**DOI:** 10.1097/MD.0000000000037969

**Published:** 2024-05-03

**Authors:** Cong Chen, Yang Liu, Songwei Yang, Ming Chen, Jing Liao

**Affiliations:** a College of Traditional Chinese Medicine, Hunan University of Chinese Medicine, Changsha, China; b Institute of Innovation and Applied Research in Chinese Medicine, Hunan University of Chinese Medicine, Changsha, China; c College of Medicine, Hunan University of Chinese Medicine, Changsha, China; d College of Pharmacy, Hunan University of Chinese Medicine, Changsha, China.

**Keywords:** bibliometric analysis, CiteSpace, familial hypertrophic cardiomyopathy, VOSviewer, Web of Science Core Collection

## Abstract

Familial hypertrophic cardiomyopathy (FHCM) is an inherited cardiac disease caused by mutations of sarcomere proteins and can be the underlining substrate for major cardiovascular events. Early identification and diagnosis of FHCM are essential to reduce sudden cardiac death. So, this paper summarized the current knowledge on FHCM, and displayed the analysis via bibliometrics method. The relevant literature on FHCM were screened searched via the Web of Science Core Collection database from 2012 to 2022. The literatures were was summarized and analyzed via the bibliometrics method analyzed via CiteSpace and VOSviewer according to topic categories, distribution of spatiotemporal omics and authors, as well as references. Since 2012, there are 909 research articles and reviews related to FHCM. The number of publication for the past 10 years have shown that the development of FHCM research has been steady, with the largest amount of literature in 2012. The most published papers were from the United States, followed by the United Kingdom and Italy. The University of London (63 papers) was the institution that published the most research articles, followed by Harvard University (45 papers) and University College London (45 papers). Keywords formed 3 clusters, focused on the pathogenesis of FHCM, the diagnosis of FHCM, FHCM complications, respectively. The bibliometric analysis and visualization techniques employed herein highlight key trends and focal points in the field, predominantly centered around FHCM’s pathogenesis, diagnostic approaches, and its complications. These insights are instrumental in steering future research directions in this area.

## 1. Introduction

Hypertrophic cardiomyopathy (HCM) is an inherited cardiac disease caused by mutations of sarcomere proteins, HCM is defined as the presence of increased LV wall thickness (with or without RV hypertrophy) or mass, that is not only explained by abnormal loading conditions.^[1]^ Diagnosis of familial HCM (FHCM) relies on abnormal thickening of the heart, however early signs of the disease are high-dynamic contraction and impaired relaxation. FHCM refers to 2 or more of the 3 generations of immediate family members who were confirmed to be HCM.^[2]^ The clinical phenotypes of patients with FHCM include the absence of cardiovascular symptoms, exertional dyspnea, chest pain, and sudden death.^[3,4]^ Therefore, early identification and diagnosis of FHCM are essential to reduce sudden cardiac death.

To date, more than 1500 mutations in genes encoding cardiac sarcomere proteins have been identified in patients with HCM.^[5,6]^ The clinical phenotypes of patients with FHCM are associated with various factors, including genotype, gene modification, and environmental influence. An initial screening studies showed that certain clinical phenotypes of patients with FHCM were possibly caused by individual gene mutations.^[7]^ Considering the correlation between them, numerous experiments have been conducted to elucidate the molecular mechanisms of the pathogenesis of HCM.

Literature is the carrier that records objectively the study of researchers. With the development of the research, numerous literature constantly expands, thus forming a relatively independent and interrelated network system. Bibliometric analysis objectively reflects the general development of the research, which is widely used to analyze and present this complex network.^[8–10]^ VOSviewer and CiteSpace are commonly used as bibliometric visualization tools.^[11,12]^

To deeply summarize the status of research and development of FHCM, this review analyzed the results of the relevant literature, and displayed the analysis via bibliometrics method, including VOSviewer and CiteSpace, based on the Web of Science Core Collection (WoSCC) database, thus providing references for the future related research.

## 2. Materials and methods

### 2.1. Collection of data

**Figure 1.**
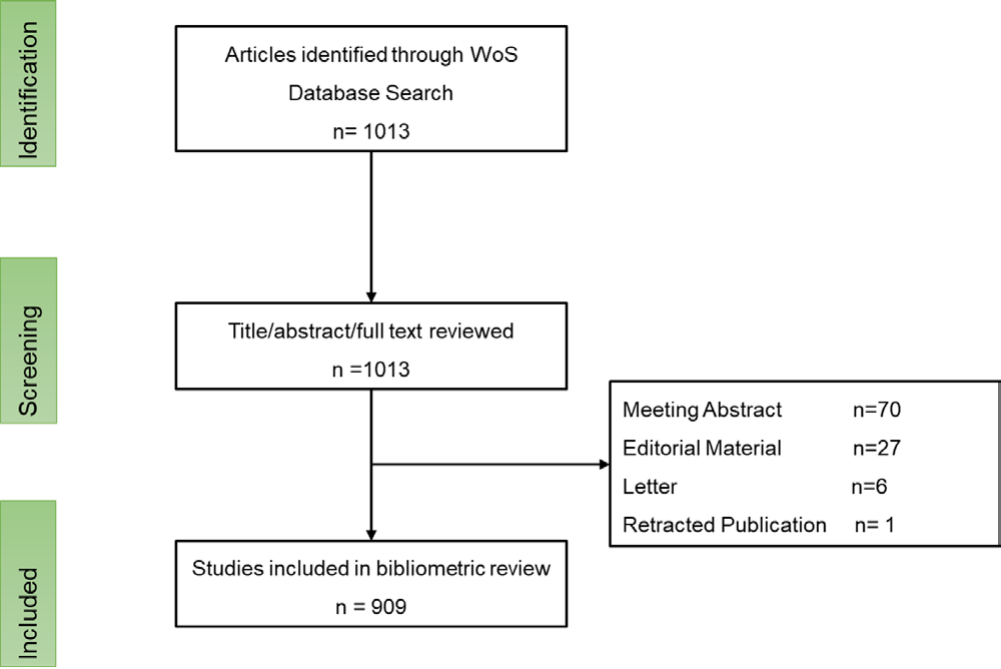
PRISMA flow diagram of the study selection process.

The literature was searched via WoSCC where keywords were followed including “Familial hypertrophic cardiomyopathy,” “FHCM,” “Hereditary hypertrophic cardiomyopathy,” “HCM.” The duration of the search was from January 1, 2012 to October 04, 2022. 1013 literatures, divided into 10 types were achieved. Therein, 67.92% of literature were research papers, and 21.62% of literatures were review papers as shown in Table 1. The other varieties of literature were conference abstracts, editorial material, book chapters, early access, proceeding papers, letter, data papers, and retracted publications, respectively. The literatures were analyzed manually one by one, and the unrelated and repeated literatures and the repeatedly published literatures were removed, such as news, conference notice, guideline and consensus. Finally, 909 literatures were included, where the quoting rate was 13,301 times, with 12,716 citing articles, 68 h-index, and 24 equally per item. A PRISMA flow diagram describing the selection method is provided in Figure 1.

**Table 1:** Top 10 productive countries regarding the researches on FHCM.

Rank	Country	Region	Quantity	Proportion/%	ACI	H-index	Total link strength
1	USA	North America	376	41.36	72.90	892	247
2	England	Western Europe	133	14.63	66.42	331	233
3	Italy	Southern Europe	91	10.01	77.96	892	173
4	China	East Asia	90	9.90	48.83	235	140
5	Germany	Central Europe	80	8.80	70.25	498	112
6	Spain	Southern Europe	52	5.72	49.21	317	105
7	Netherlands	Western Europe	49	5.39	91.08	499	104
8	Canada	North America	48	5.28	86.81	317	70
9	Japan	East Asia	40	4.40	49.70	187	64
10	Australia	Oceania	39	4.29	42.87	80	59

ACI = average citations per item, FHCM = familial hypertrophic cardiomyopathy.

### 2.2. Analysis of data

Nine hundred nine documents were analyzed by Excel (v.2019), VOSviewer (v.1.6.18), and CiteSpace (v.6.1.R2). Due to its excellent ability of processing graphs and a huge number of data, VOSviewer is applied in reasonable networks for data visualization as a bibliometric software.^[13]^ CiteSpace, a Java-based software, is gradually developed for visual analysis of bibliometrics.^[14]^ In this study, temporal distributions of FHCM-associated studies with high cited times in the past 10 years were analyzed through Excel. Visual maps including research institutions, country/regions, disciplines as well as authors were conducted through VOSviewer. The research hotspots and frontiers of the FHCM were predicted and displayed visually through CiteSpace.

### 2.3. Research ethics

Ethical approval was not required, as the data used in this article were downloaded from the public databases and did not involve interaction with human participants.

## 3. Results

### 3.1. Map of temporal distribution

**Figure 2.**
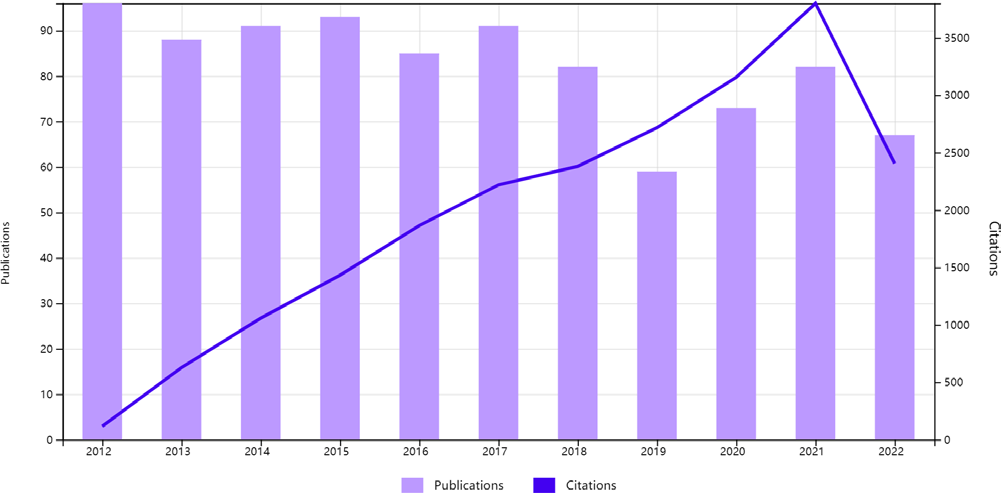
Trends of publications and numbers of cited papers from 2012 to 2022 throughout the world.

The numbers of literature in a period reflect trends and speed of the research development.^[15]^ The publication numbers in the last 10 years showed that the development of FHCM researches were as steady, with the largest amount of literature in 2012. Compared with 2018, the number of published papers significantly declined in 2019. The cited times is an important index to quantitatively evaluate academic quality. As shown in Figure 2, from 2012 to 2021, the cited times increased every year, with the highest quote in 2021. In 2022, although lower than 11 months were counted, the tendency showed that quoted rate would exceed that in 2021.

### 3.2. Distribution of country/region

As shown in Table 1, the most published papers were from the United States, followed by the United Kingdom and Italy, accounting for 41.36%, 14.63%, and 10.01% of the total reports, respectively. These countries accounted for more than half of the total number of studies reported, which implies that they are very interested in FHCM research. Studies from the Netherlands (91.08), Canada (86.81), and Italy (77.96) had the highest mean citations per item (ACI), demonstrating that these countries initiated studying FHCM earlier, and their results were relatively mature.

**Figure 3.**
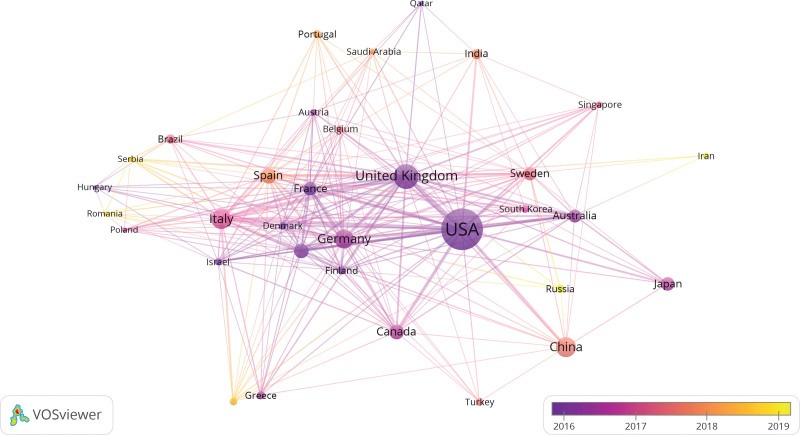
Map of cooperation among countries with FHCM. FHCM = familial hypertrophic cardiomyopathy.

Figure 3 showed the condition of cooperation among countries. Parameters of VOSviewer were followed with Linlog/modularity, the minimum number of a country with 5. The achieved results were from 63 countries, with 33 countries meeting the thresholds. In terms of cooccurrence analysis, countries were classified into diverse clusters and colors according to the time when they appeared, adding time into the network of co-occurrence countries. Diverse colors represented the year when countries appeared. The darker purple represented the earlier appearance of the country, whereas the darker yellow represented the later appearance (Fig. 3). The USA mainly cooperated with Britain, Canada, Germany, and Australia, whereas England mainly cooperated with America, Germany, the Netherlands as well as Italy. And Italy frequently cooperated with USA, Germany, Canada, UK, France, and Spain.

### 3.3. Authors distribution and research institutions

Tardiff, Jil C. from the University of Arizona had the most published articles, followed by Elliott, Perry from University College London in England and Olivotto, lacopo from the University of Florence in Italy (Table 2). Six of the top 10 authors came from the USA.

**Table 2:** Top 10 authors in the studies of FHCM.

Rank	Author	Country	Institution	TP	ACI	H-index
1	Tardiff, Jil C.	USA	University of Arizona	19	65.00	176
2	Elliott, Perry	England	University College London	19	38.26	133
3	Olivotto, lacopo	Italy	University of Florence	16	50.13	110
4	Seidman, Christine E.	USA	Harvard Medical School	15	48.67	140
5	Szczesna-Cordary, Danuta	USA	University of Miami	15	55.27	136
6	Ho, Carolyn Y.	USA	Brigham & Women’s Hospital	13	48.23	96
7	Kimura, Akinori	Japan	Tokyo Medical & Dental University	12	40.83	144
8	Semsarian, C.	Australia	University of Sydney	12	37.08	55
9	Kazmierczak, Katarzyna	USA	University of Miami	12	55.75	136
10	Sadayappan, Sakthivel	USA	University of Cincinnati	11	82.18	294
11	Carrier, Lucie	Germany	University Medical Center Hamburg-Eppendorf	11	83.82	154

ACI = average citations per item, FHCM = familial hypertrophic cardiomyopathy, TP = total publications.

**Figure 4.**
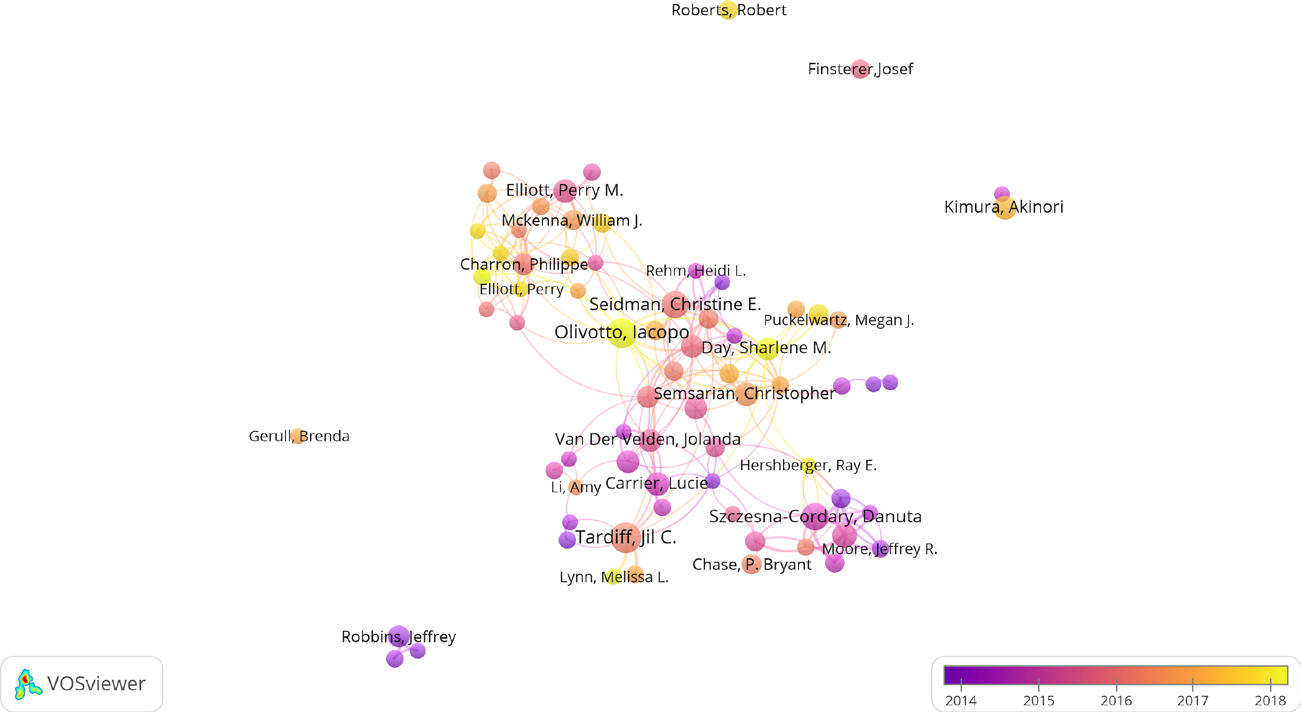
VOSviewer visualization map of authors in the studies of FHCM. FHCM = familial hypertrophic cardiomyopathy.

In Figure 4, different clusters represented the condition of cooperation among authors. Parameters of VOSviewer were followed with Linlog/modularity, the minimum number of an author with 5. And 73 authors met the thresholds of total 505. In terms of co-occurrence analysis, authors were classified into diverse clusters and colors according to the time when they appeared, adding time into the network of co-occurrence authors. Tardiff, Jil C., and Schwartz Steven D., Carrier, Lucie, Van der Velden, Jolanda, Olivotto, Lacopo, Day, Sharlene M. closely cooperated. Olivotto, lacopo worked closely with Tardiff, Jil C., Van der Velden, Jolanda, Charron, Philippe, Day, Sharlene M., Semsarian, and Christopher. Elliott, Perry worked closely with Mckenna, William J., Charron, Philippe. Szczesna-Cordary, Danuta worked closely with Wang, Li, Hershberge, Ray E, Liang, and Jingsheng.

In Table 3, the University of London (63 papers) was the institution that published the most research articles, followed by Harvard University (45 papers) and University College London (45 papers). The University of Arizona had the highest ACI (99.00), which was closely followed by the University of California with an ACI of 96.29, and Imperial College London with an ACI of 78.71.

**Table 3:** Top 10 institutions in the studies of FHCM.

Rank	Institution	Country	Quantity	SOTC	ACI	H-index
1	University of London	England	63	4198	66.63	331
2	Harvard University	USA	45	2836	63.02	317
3	University College London	England	45	3182	70.71	331
4	University of Arizona	USA	38	3762	99.00	781
5	University of California system	USA	38	3659	96.29	892
6	Harvard medical school	USA	36	2374	65.94	317
7	Imperial Coll Ege London	England	35	2755	78.71	331
8	Institut national De La Santeet De La	Germany	31	1705	55	317
9	Recherche medicale INSERM Brigham womens hospital	USA	29	1953	67.34	317
10	Udice French Research Universities	Germany	29	1786	61.59	317

ACI = average citations per item, FHCM = familial hypertrophic cardiomyopathy, SOTC = sum of times cited.

**Figure 5.**
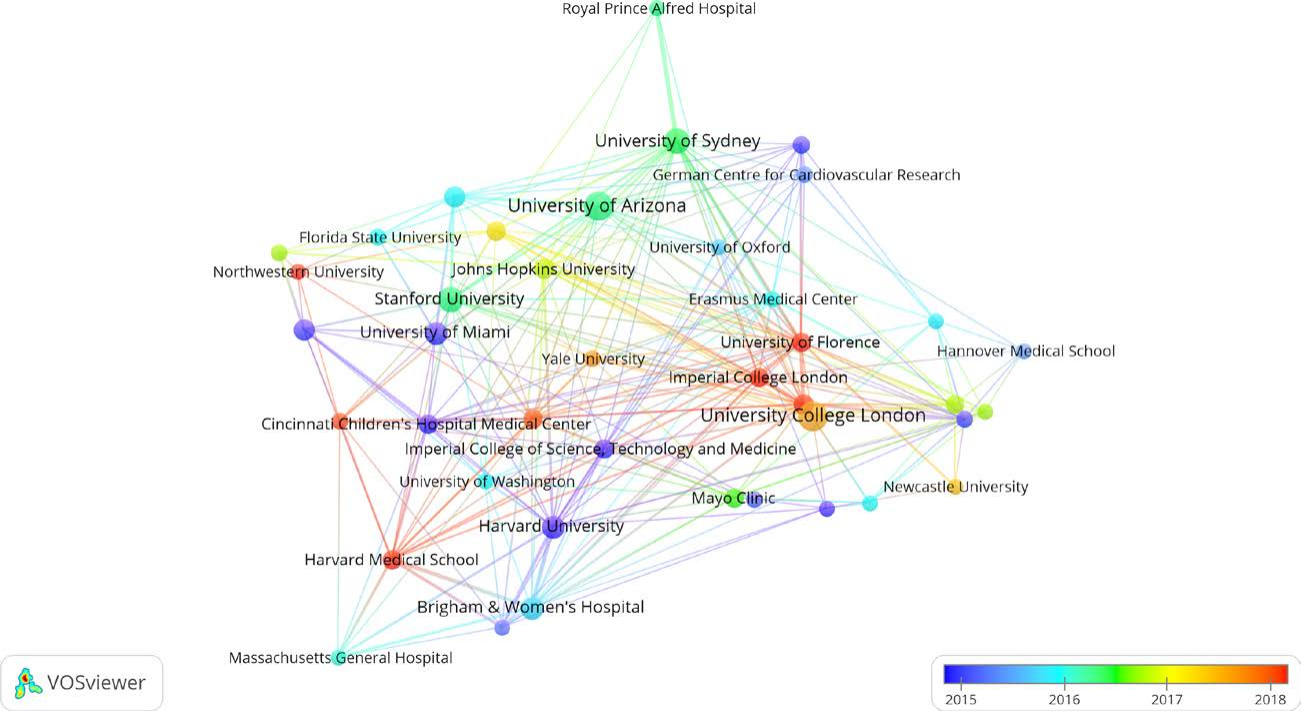
Map of VOSviewer visualization of institutions in the studies of FHCM. FHCM = familial hypertrophic cardiomyopathy.

Parameters of VOSviewer were followed with Linlog/modularity, the minimum number of an institution with 10. Of the 1509 institutions, 41 met the threshold. In terms of analysis of co-occurrence, institutions were classified into diverse clusters and colors according to the time when they appeared, adding time into the network of co-occurrence institutions. Diverse colors represented the year when institutions appeared. The darker blue represented earlier appearance of FHCM research in the institution, whereas the darker red represented later appearance (Fig. 5).

As shown in Figure 5, University College London, Imperial College of Science, Technology and Medicine, University of Cincinnati, Stanford University, Johns Hopkins University, Ohio State University, University of Sydney, Imperial College London, University of Florence, Heidelberg University, King’s College London, Yale University, University of Amsterdam closely collaborated. University of Arizona, University of Sydney, University of Oxford, Johns Hopkins University, Stanford University, German Centre for Cardiovascular Research, University of Florence, Erasmus Medical Center, Heidelberg University, Careggi University Hospital, University of Washington closely collaborated. Stanford University, University of Cincinnati, Ohio State University, Northwestern University, University of Michigan, University of Washington, Yale University, University of Arizona, University of Sydney, Erasmus Medical Center, University of Florence, Imperial College London, University College London, Harvard Medical School, Harvard University closely collaborated.

### 3.4. Distribution of disciplines and journals

In the number of published papers, the top 3 disciplines were cardiac cardiovascular systems, genetics heredity, and biochemistry molecular biology (Table 4). The other associated subjects with literature included cell biology (9.02%), physiology (7.26%), medicine general internal (6.27%), medicine research experimental (5.39%), peripheral vascular disease (4.84%), multidisciplinary sciences (3.85%), biophysics (3.74%), and pediatrics (3.41%).

**Table 4:** categories within the top 20 in the studies of FHCM.

Rank	Quantity	WoSCC categories	Percentage/%
1	365	Cardiac cardiovascular systems	40.15
2	124	Genetics heredity	13.64
3	116	Biochemistry molecular biology	12.76
4	82	Cell biology	9.02
5	66	Physiology	7.26
6	57	Medicine general internal	6.27
7	49	Medicine research experimental	5.39
8	44	Peripheral vascular disease	4.84
9	35	Multidisciplinary sciences	3.85
10	34	Biophysics	3.74
11	31	Pediatrics	3.41
12	22	Neurosciences	2.42
13	19	Veterinary sciences	2.09
14	18	Hematology	1.98
15	16	Clinical neurology	1.76
16	14	Pathology	1.54
17	13	Biotechnology applied microbiology	1.43
18	13	Chemistry multidisciplinary	1.43
19	12	Endocrinology metabolism	1.32
20	12	Radiology nuclear medicine medical imaging	1.32

FHCM = familial hypertrophic cardiomyopathy, WoSCC = Web of Science Core Collection.

As shown in Table 5, the Journal of Molecular and Cellular Cardiology had the highest number of publications toward on FHCM, followed by the Frontiers in Physiology (20 articles), Journal of Muscle Research and Cell Motility (17 articles), Cardiovascular Research (16 articles), Circulation Research (15 articles). International Journal of Molecular Sciences (167.85) had the highest ACI, followed by the American Journal of Physiology Heart and Circulatory Physiology (123.00), Circulation Research (109.87), Cardiovascular Research (102.31), Journal of the American College of Cardiology (87.55), and Journal of Muscle Research and Cell Motility (80.41).

**Table 5:** Journals within top 20 the studies of FHCM.

Rank	Quantity	Journal	ACI	IF (2022)	JCR
1	21	*Journal of Molecular and Cellular Cardiology*	53.33	5.763	Q2/Q3
2	20	*Frontiers in Physiology*	59.25	4.755	Q1
3	17	*Journal of Muscle Research and Cell Motility*	80.41	3.352	Q3
4	16	*Cardiovascular Research*	102.31	13.081	Q1
5	15	*Circulation Research*	109.87	23.213	Q1
6	13	*European Heart Journal*	45.00	35.855	Q1
7	13	*International Journal of Molecular Sciences*	167.85	6.208	Q1
8	13	*Journal of Biological Chemistry*	65.00	5.486	Q1
	12	*Archives of Biochemistry and Biophysics*	53.25	4.114	Q2
9	12	*Circulation*	49.00	39.918	Q1
10	12	*Circulation Cardiovascular Genetics*	40.75	4.534	Q1
11	12	*PloS One*	51.67	3.752	Q2
12	11	*American Journal of Physiology Heart and Circulatory Physiology*	123.00	5.125	Q2
13	11	*Journal of the American College of Cardiology*	87.55	27.203	Q1
14	11	*Journal of the American Heart Association*	38.00	6.106	Q2
15	11	*Pflugers Archiv European Journal of Physiology*	73.27	4.458	Q2
16	11	*Proceedings of the National Academy Sciences of the United States of America*	54.36	12.779	Q1
17	10	*Heart*	45.50	7.365	Q1
18	10	*Revista Portuguesa De Cardiologia*	32.50	1.651	Q4
19	9	*American Journal of Cardiology*	24.67	3.133	Q3
20	9	*Biophysical Journal*	61.67	3.699	Q2
21	9	*Circulation Journal*	42.78	3.350	Q3

FHCM = familial hypertrophic cardiomyopathy, IF = impact factor, JCR = journal citation reports.

### 3.5. Highly cited literatures

The cited times of literature is a crucial indicator to evaluate quantitatively academic quality. The top 10 highly cited literature was summarized in Table 6. The article “Truncations of Titin Causing Dilated Cardiomyopathy”^[16]^ was the paper with the highest cited times. Herman, Daniel S. et al confirmed that sarcomere abnormalities can characterize both HCM and Coronary microvascular dysfunction. The article “Genetics of Hypertrophic Cardiomyopathy After 20 Years Clinical Perspectives”^[17]^ was the second most cited paper. This paper conducted by Maron, Barry J. demonstrated that genetic testing played an important role in the early screening, diagnosis, treatment and prognosis prediction of HCM. The accuracy of genetic testing reports is the key to translating basic genetic information into indicators of reference for HCM patients. The article “Abnormal Calcium Handling Properties Underlie Familial Hypertrophic Cardiomyopathy Pathology in Patient-Specific Induced Pluripotent Stem Cells” was the thirdly most cited paper.^[18]^ Lan et al demonstrated that Arg663His-mediated elevation of Ca^2+^ was an important mechanism of arrhythmias. Therefore, the elevation of Ca^2+^ at the single-cell level was a priming factor for the diagnosis of HCM.

**Table 6:** Top 10 highly cited literatures.

Rank	Title	Journal	Type	Author	Year	SOTC
1	Truncations of titin causing dilated cardiomyopathy^[16]^	*New England Journal of Medicine*	Article	Herman, Daniel S.	2012	793
2	Genetics of hypertrophic cardiomyopathy after 20 years clinical perspectives^[17]^	*Journal of the American College of Cardiology*	Review	Maron, Barry J.	2012	428
3	Abnormal calcium handling properties underlie familial hypertrophic cardiomyopathy pathology in patient-specific induced pluripotent stem cells^[18]^	*Cell Stem Cell*	Article	Lan, Feng	2013	416
4	Drug screening using a library of human induced pluripotent stem cell-derived cardiomyocytes reveals disease-specific patterns of cardiotoxicity^[19]^	*Circulation*	Article	Liang, P	2013	354
5	Atlas of the clinical genetics of human dilated cardiomyopathy^[20]^	*European Heart Journal*	Article	Haas, J	2015	323
6	Genetics of human cardiovascular disease^[21]^	*Cell*	Review	Kathiresan, Sekar	2012	271
7	Results of clinical genetic testing of 2912 probands with hypertrophic cardiomyopathy: expanded panels offer limited additional sensitivity^[22]^	*Genetics in Medicine*	Article	Alfares, Ahmed A.	2015	267
8	Structure of the rigor actin-tropomyosin-myosin complex^[23]^	*Cell*	Article	Behrmann, Elmar	2012	245
9	Diagnostic work-up in cardiomyopathies: bridging the gap between clinical phenotypes and final diagnosis. A position statement from the ESC Working Group on Myocardial and Pericardial Diseases^[24]^	*European Heart Journal*	Article	Rapezzi, Claudio	2013	230
10	Calcium signaling and cardiac arrhythmias^[25]^	*Circulation Research*	Review	Landstrom, Andrew P.	2017	221

SOTC = sum of times cited.

For article types, the most cited literatures included 7 articles and 3 reviews. The publication time of highly cited literatures were from 2012 to 2015.

### 3.6. Co-cited references

**Figure 6.**
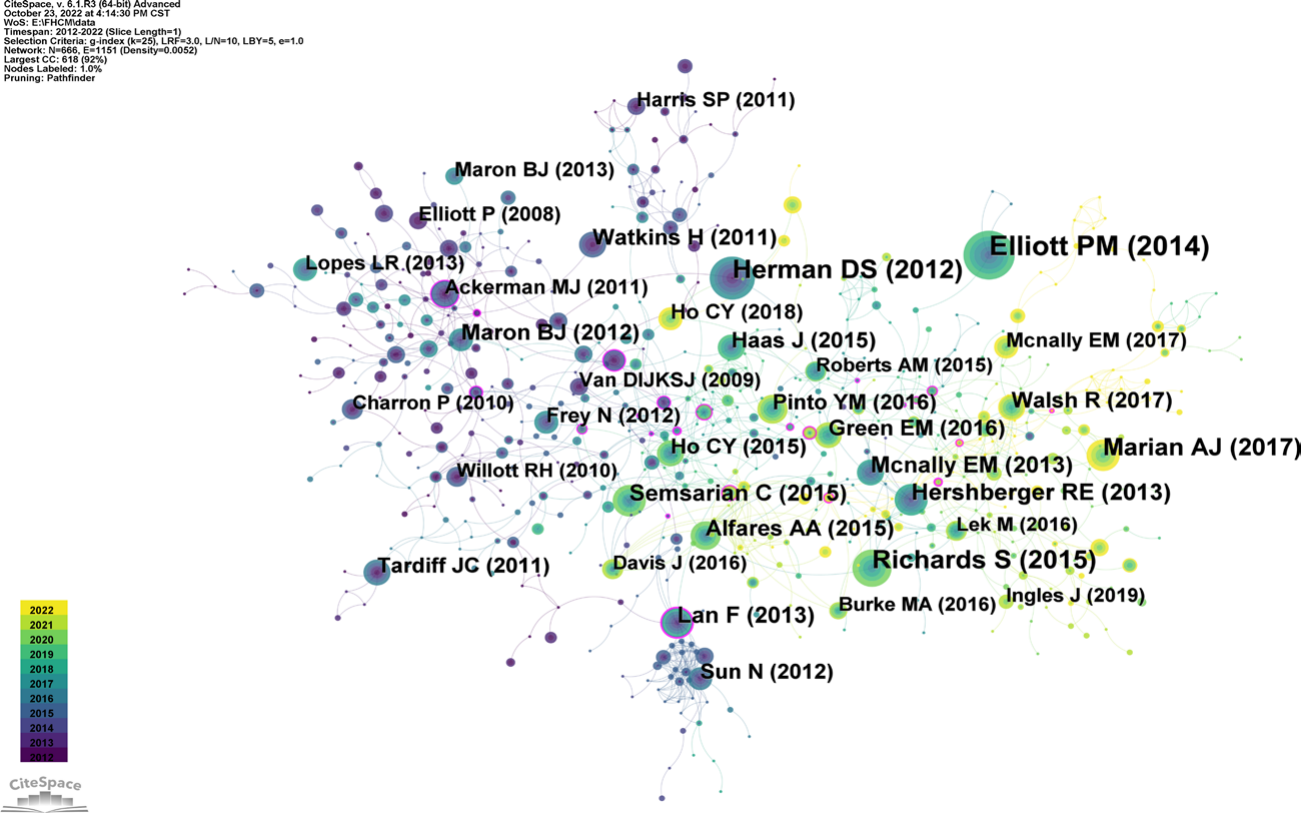
Co-citation network of references.

Parameters of CiteSpace were followed as duration with 2012 to 2022, years per slice with 1, node type with cited reference, selection criteria with g-index, K = 25. According to the parameters, a network with 666 nodes, 1151 connections, and 0.52% density was achieved (Fig. 6). Modularity Q was 0.84, and Silhouette S was 0.95. Network modularity is commonly evaluated by the index of modularity. The higher value of a network represented the better clustering. The value space of Q with [0,1], and Q above 0.3 indicated that the structure of the network community was significant.^[13]^ Silhouette was evaluated by measuring network homogeneity. The Silhouette value was closer to 1 represented representing the higher network homogeneity. Clustering was considered reasonable when the value was above 0.5.^[13]^ The result of clustering with a value of 0.8 was highly reliable. In Figure 6, the network community structure was credible, and had a high homogeneity and reliability. The nodes marked with purple circles represented high centrality (Fig. 6), which were used in CiteSpace to evaluate the significance of documents and achieved the top 10 co-cited references (Table 7). In the thirdly cited article, 2 references appeared in the reference list through co-citation analysis, indicating that these references formed a co-citation relevance.^[33]^

**Table 7:** Top 10 co-cited references.

Rank	Frequency	Centrality	Title	Journal	Author	Year
1	73	0.01	2014 ESC Guidelines on diagnosis and management of hypertrophic cardiomyopathy: the Task Force for the Diagnosis and Management of Hypertrophic Cardiomyopathy of the European Society of Cardiology (ESC)^[26]^	*European Heart Journal*	Elliott PM	2014
2	59	0.07	Truncations of titin causing dilated cardiomyopathy^[16]^	*New England Journal of Medicine*	Herman DS	2012
3	51	0.08	Standards and guidelines for the interpretation of sequence variants: a joint consensus recommendation of the American College of Medical Genetics and Genomics and the Association for Molecular Pathology^[27]^	*Genetics in Medicine*	Sue Richards	2015
4	40	0	Hypertrophic cardiomyopathy: genetics, pathogenesis, clinical manifestations, diagnosis, and therapy^[28]^	*Circulation Research*	Ali J Marian	2017
5	35	0.04	Dilated cardiomyopathy: the complexity of a diverse genetic architecture^[29]^	*Nature Reviews Cardiology*	Ray E Hershberger	2013
6	31	0.05	New perspectives on the prevalence of hypertrophic cardiomyopathy^[30]^	*Journal of the American College of Cardiology*	Christopher Semsarian	2015
7	31	0.01	Genetic mutations and mechanisms in dilated cardiomyopathy^[31]^	*Journal of Clinical Investigation*	Elizabeth M McNally	2013
8	31	0.24	Abnormal calcium handling properties underlie familial hypertrophic cardiomyopathy pathology in patient-specific induced pluripotent stem cells^[18]^	*Cell Stem Cell*	Feng Lan	2013
9	30	0.10	Results of clinical genetic testing of 2912 probands with hypertrophic cardiomyopathy: expanded panels offer limited additional sensitivity^[22]^	*Genetics in Medicine*	Ahmed A Alfares	2015
10	30	0	Inherited cardiomyopathies^[32]^	*New England Journal of Medicine*	Hugh Watkins	2011

The paper “2014 ESC Guidelines on diagnosis and management of hypertrophic cardiomyopathy: the Task Force for the Diagnosis and Management of Hypertrophic Cardiomyopathy of the European Society of Cardiology (ESC),”^[26]^ had the highest cited frequency. It is a guideline document, which summarized FHCM and gave physicians advice, including epidemiology, etiology, diagnosis, genetic testing, control of symptoms and complications, recommendations for routine follow-up, and special issues. “Truncations of titin causing dilated cardiomyopathy”^[16]^ was the second most cited and “Standards and guidelines for the interpretation of sequence variants: a joint consensus recommendation of the American College of Medical Genetics and Genomics and the Association for Molecular Pathology” was the third most cited.

In CiteSpace, mediation centrality nodes above 0.1 become the key points. In Table 7, the betweenness centrality of “Abnormal calcium handling properties underlie familial hypertrophic cardiomyopathy pathology in patient-specific induced pluripotent stem cells”^[18]^ was 0.24. In Tables 7 and 8, references with high citation frequency and high co-citation frequency all contain these 3 references.^[16,18,26]^

**Table 8:** Keywords within the top 20 in the studies of FHCM.

Rank	Keywords	Occurrences	Total link strength	Rank	Keywords	Occurrences	Total link strength
1	Hypertrophic cardiomyopathy	371	1653	11	European-society	67	390
2	Familial hypertrophic cardiomyopathy	302	1252	12	Mouse model	75	362
3	Cardiomyopathy	224	1113	13	Prevalence	68	362
4	Mutation	214	1067	14	Gene	73	358
5	Dilated cardiomyopathy	174	905	15	Task-force	58	342
6	Heart failure	130	698	16	Sudden cardiac death	61	329
7	Diagnosis	113	587	17	Alpha-tropomyosin	61	327
8	Genetics	101	557	18	Guidelines	47	294
9	Disease	92	477	19	Right-ventricular cardiomyopathy	48	282
10	Binding protein-c	72	392	20	Phosphorylation	50	276

FHCM = familial hypertrophic cardiomyopathy.

**Figure 7.**
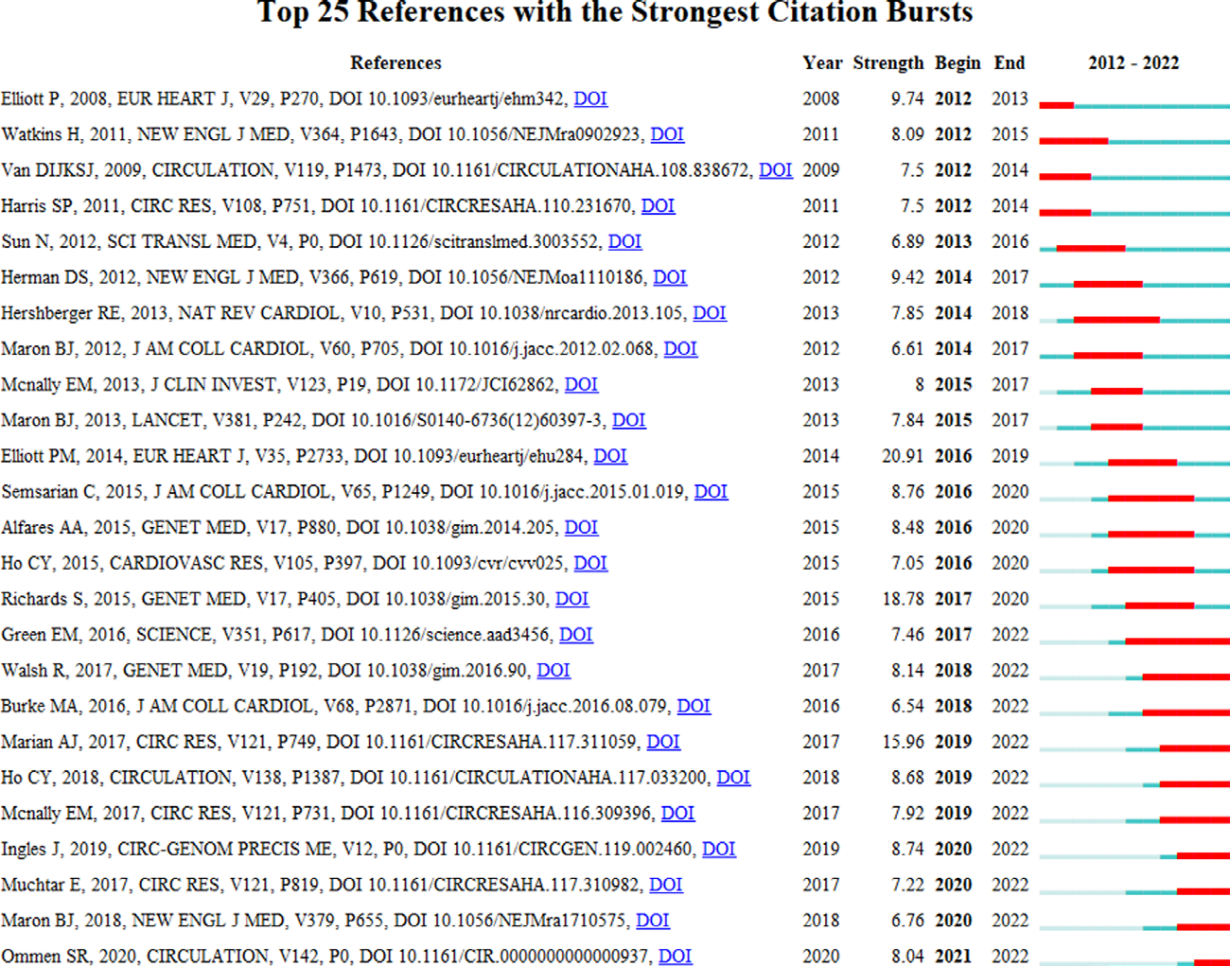
References within the top 20 with the strongest citation times.

Figure 7 showed the references of the top 25 with the highest citated times. The article “2014 ESC Guidelines on diagnosis and management of hypertrophic cardiomyopathy: the Task Force for the Diagnosis and Management of Hypertrophic Cardiomyopathy of the European Society of Cardiology (ESC)” with the highest citation was published in 2016. This article had an intensity of 20.91.

### 3.7. Analysis of research hotspots and frontier

#### 3.7.1. Analysis of research hotspots

**Figure 8.**
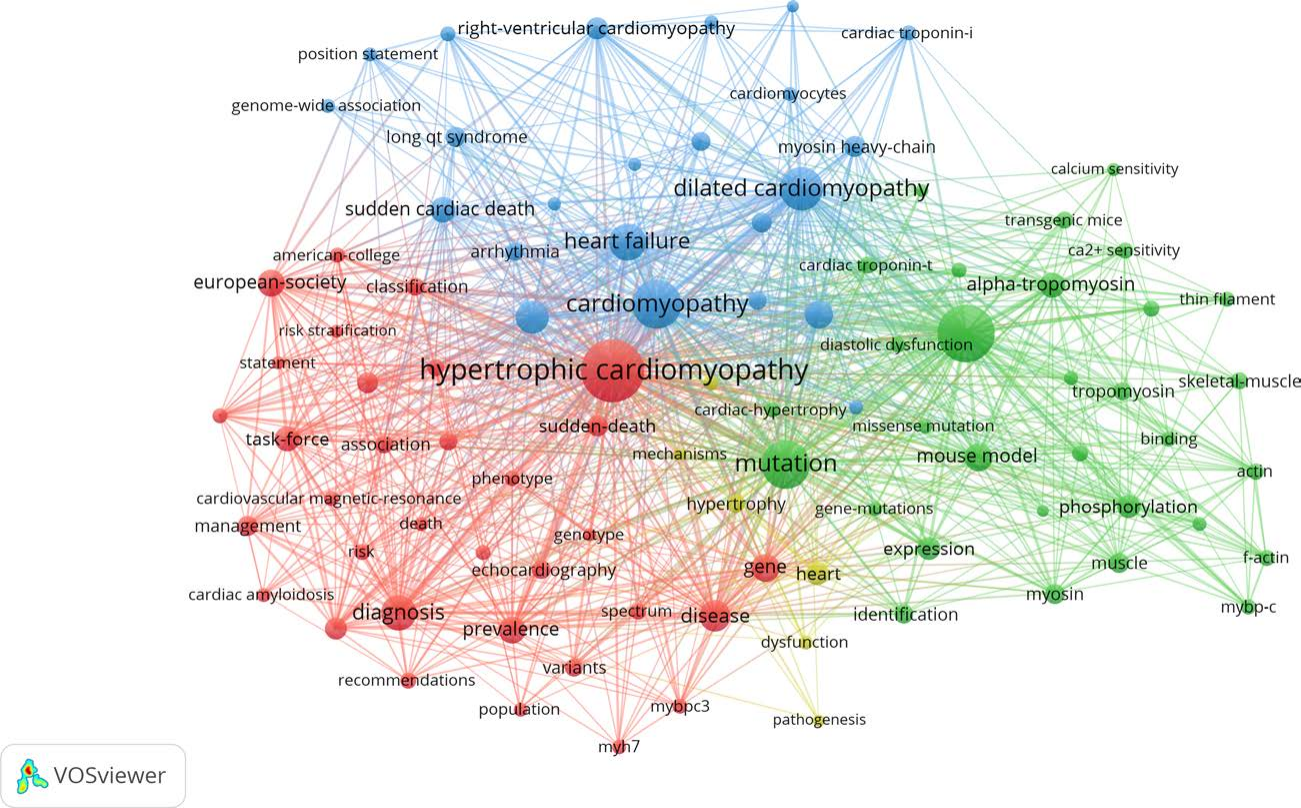
Map of keyword clustering in the studies of FHCM. FHCM = familial hypertrophic cardiomyopathy.

Keywords are central and essential in a paper, which represents the hotspots of a research field. In this study, key co-occurrence analysis was used to determine the hotspots and uncover the development of researches on FHCM. In Table 8, hypertrophic cardiomyopathy, familial hypertrophic cardiomyopathy, cardiomyopathy, dilated cardiomyopathy, heart failure, and sudden cardiac death were shown. The more frequent keywords were mutation, diagnosis, genetics, binding protein-c, European- society, mouse model, prevalence, gene, task-force, and alpha-tropomyosin. VOSviewer parameters were followed as the method with Linlog/modularity, the minimum number of occurrences of a keyword with 15. There were 5990 keywords where 93 keywords met the thresholds. The strength of co-occurrence links for each 93 keywords with other keywords was calculated. Keywords with the greatest total link strength were selected. The co-occurrence network graph of keywords showed that the thicker connection between nodes indicating indicates the higher frequency of 2 keywords (Fig. 8). Keywords formed 3 clusters, representing the main research directions in the FHCM field.

The red cluster mainly focused on the pathogenesis of FHCM. Keywords were mutation, mouse model, expression, actin, calcium sensitivity, binding-protein-c, cardiac myosin, dysfunction and contraction. The green cluster mainly focused on the diagnosis of FHCM with keywords including diagnosis, association, classification, genetic testing, gene type, risk, risk stratification, prevalence, population, variants, spectrum and long qt syndrome. The blue cluster was associated with FHCM complications. The specifically related diseases were arrhythmia, heart failure, sudden cardiac death, dilated cardiomyopathy, and restrictive cardiomyopathy.

#### 3.7.2. Identification of research frontiers

**Figure 9.**
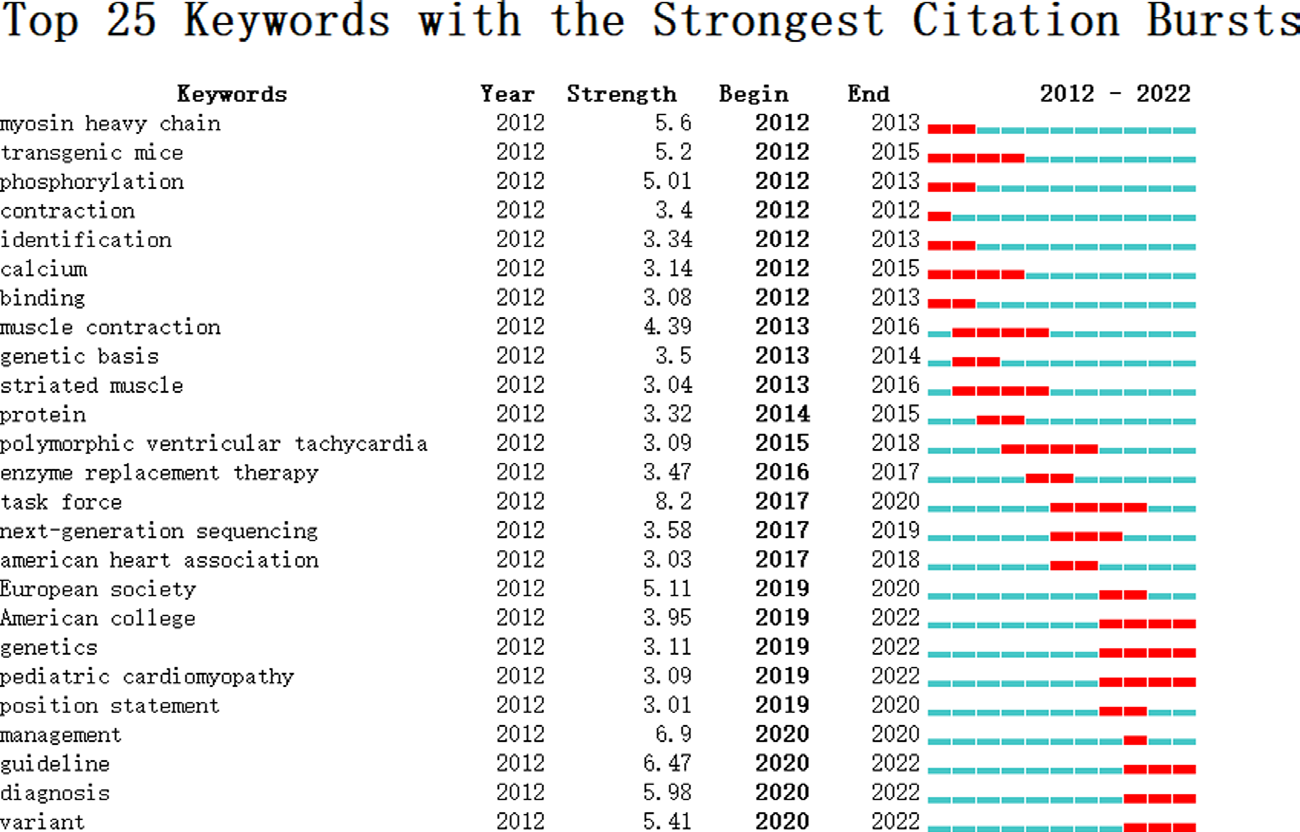
Top 15 keywords with the strongest citation bursts.

In terms of the co-occurrence network of keywords, emergent word detection of keywords was conducted. The top 25 keywords with the highest citated times toward FHCM researches within the last 10 years were shown. In Figure 9, the blue line denoted the time axis while the red segment appeared as the burst detection, indicating the year of initiation and termination, and burst duration. Notably, “task force” had the highest quoted times, followed by management, guideline, diagnosis, myosin heavy chain, variant, transgenic mice, European society, phosphorylation, and muscle contraction. In terms of the start time of emergence, it was shown that myosin heavy chain, transgenic mice, phosphorylation, contraction, identification, calcium, binding, muscle contraction, genetic basis, and striated muscle were achieved attention in the past 10 years. Guideline, diagnosis, and variant have been the current research frontier in FHCM.

## 4. Discussion

### 4.1. General information

Based on the numbers of publications, the development of FHCM researches was at a stable stage. In 2012, the number of articles published was the highest, possibly due to the increased pathophysiology of FHCM. During this period, Herman et al^[16]^ confirmed that 25% of patients with idiopathic dilated cardiomyopathy and 18% of patients with dilated cardiomyopathy were caused by TTN truncated mutation, simultaneously defined defining its functional effects. Incorporating sequencing methods to detect TTN truncation into genetic testing could significantly improve the sensitivity of the test, allowing for early diagnosis and therapeutic intervention for many patients with dilated cardiomyopathy. Additionally, the cited times of related literature toward on FHCM increased year by year, indicating the continuous attention to this field.

The researches on FHCM started in developed countries, indicating that the related studied in these countries have been relatively sophisticated. University of London, Harvard University, and University College London have conducted numerous studies with high quality on FHCM. In terms of studies numbers, the USA was the country which had the largest number of relevant studies. For the quantity and quality, developed countries have more abundant researches compared with developing countries and underdeveloped countries.^[34]^ The root cause of this phenomenon may be uneven economic development. First, genetic testing is the “gold standard” for FHCM diagnosis, but due to the high costs associated with genetic testing, it is difficult to afford in low-income countries. Furthermore, although HCM is an inherited cardiomyopathy, an underlying genetic cause of the disease has been found in only 34% of patients.^[35]^ Immediate family members of HCM patients should be screened for the disease.^[36]^ Patients who are most likely to have positive genetic test results are those who are <45 years old, have a family history of HCM, a family history of sudden cardiac death, have an inverted nasal septum shape curve, and have no systemic hypertension.^[35]^ This combination of high economic costs and low diagnostic efficiency has led to a refusal in low-income countries to use genetic testing to identify and classify patients with FHCM. Secondly, the difference in economic level leads to the difference in the level of national education and the level of national medical knowledge. People in less developed areas did not seek the cause of death despite the sudden death due to FHCM. Moreover, developed countries have built a better medical management system, carried out continuous active management of diseases, and built an evidence-based intelligent platforms to help medical staff and patients keep track of their conditions. In addition, developed countries have better laboratories and clinical centers, which are more conducive to the mechanism research of FHCM.

In terms of regional differences, FHCM related studies in western countries are more mature and rich compared with eastern countries. It is worth noting that China is among the top 10 countries with the largest number of studies on FHCM. It is one of the developing countries as well as one of the Asian countries. This phenomenon indicates that FHCM research has been paid more and more attention in developing countries and Asian countries. Except for close cooperation between European countries, there is relatively little cooperation between other countries, and inter-agency cooperation is mostly carried out domestically. Among research institutions, the University of Arizona has the highest ACI. In short, it is imperative to strengthen international cooperation, learn from advanced medical management systems of developed countries, promote the research and development of FHCM, and achieve a global win-win situation.

The h-index is an important indicator to quantify an individual’s scientific research output.^[37]^ Professor Sadayappan Sakthivel of the University of Cincinnati published the highest H-index, and he published many articles with high quality on FHCM in various institutions, including the University of Cincinnati, Loyola University Chicago, and Cincinnati Children’s Hospital Medical Center, which all in the USA. This phenomenon confirmed the results of our study, in which United States had advantages in researches on FHCM with quantity and quality.

We considered that the communications of FHCM between disciplines were extensive. The related disciplines included cardiovascular system, genetics heredity, biochemistry molecular biology, cell biology, and physiology. Interdisciplinary research is helpful to understand FHCM from different perspectives. Journal Citation Reports are is an important index to evaluate the quality of journals. Papers published in journals classified in Q1 had higher academic influence and credibility.^[38]^ The journals in the top 20 with the highest ACI were mostly in Q1 or Q2, indicating the strong academic influence on FHCM researches. Impact factors are also key indicators to measure journals quality.^[39]^ Generally, journals with higher impact factors had higher academic value. Circulation (IF39.918, Q1), European Heart Journal (IF35.855, Q1) and Journal of the American College of Cardiology (IF 27.203, Q1) had an impact factor value >25, which were counted by Journal Citation Reports in 2021. This indicated that these journals published studies with convincing results.

The top 10 cited literature mainly focused on the genetic research of FHCM, mainly carrying out the research of gene mutation, genetic map, and gene diagnosis of FHCM. Therefore, genetic diagnosis and genetic mapping of patients with FHCM were the centers of current research. It also showed the importance of guidelines in academical and clinical researches. The literature with the highest citated frequency and co-citation frequency were essential in the FHCM researches. However, citations did not fully decide the academic level of articles, because of earlier published articles usually have higher citations.^[40]^

### 4.2. The hotspots and frontiers

In terms of keyword cluster and time line, current research hotspots of FHCM were mainly including included pathogenesis, diagnosis, and complications, in which genes played an important role in them. The genetic basis of FHCM mainly originated from the mutation of fibroid protein, and 15 loci were identified. However, the underlying mutations are still unknown, and less than half of the associated genes at these sites were identified. The researchers conducted a series of studies on pathogenic gene mutation sites in patients with FHCM. In 1999, missense mutations in the lamin A/C gene bar domain were found to be the cause of dilated cardiomyopathy.^[41]^ Its function was unclear, and it was possibly not related to mechanical forces.^[42]^ Several mutations of troponin T and beta-myosin heavy chain were recognized to be responsible for FHCM.^[43,44]^ To date, more than 1500 mutations in genes encoding myocardial ganglion proteins have been identified in HCM patients,^[5,6]^ such as thick filament (beta MyHC), thin filament (cTnT and Tm), and associated proteins (MyBP-C). However, even in patients with a family history, the detection rate of clinical genetic testing for HCM is not more than 60%. In addition, the clinical features of FHCM are highly heterogeneous, including disease severity, age of onset, and disease progression.^[45]^ The unstable penetrance of HCM family members, and insufficient genotype-phenotype correlation led to difficulty in predicting diseases severity.^[46]^ Truncated variants of alpha protein kinase 3 were identified as a cause of autosomal dominant hypertrophic cardiomyopathy, which was characterized by a severe cardiac phenotype with extensive myocardial fibrosis and progression to heart failure.^[47]^ Above all, identifying genetic mutations of FHCM, exploring the relationship between clinical variability and genetic heterogeneity, early diagnosis of patients, risk identification, and using implantable defibrillator implement were essential for personalized, accurate health care and intervention.^[48,49]^

### 4.3. Limitations

This study still had some limitations. Firstly, this study analyzed articles from the WoSCC database, but not involved other databases such as PubMed and Scopus. Secondly, CiteSpace and VOSviewer were not able to completely displace system retrieval. For instance, the literature was collected from 2012 to 2022. The numbers of studies were different from the actual numbers due to continuous updating of WoSCC documents, thus citations and h-index accordingly changing. Thirdly, although all literature of on FHCM was collected from WoSCC database, the credibility of the analysis was possibly not good, due to the different quality among the studies.

## 5. Conclusions

In this study, research progress, hotspots, and frontiers toward FHCM in the past 10 years were elucidated through information visualization technology. The results demonstrated investigations of FHCM, and provided the potential research partners. The development of FHCM researches have been stable and the cited times increased each year. More FHCM researches have been conducted in developed countries, compared with developing countries. It is necessary to strengthen international collaboration and communication. The scholars, institutions, relevant journals, and representative literatures were identified to be important. And researches mainly focused on the pathogenesis, diagnosis, and complications of FHCM.

## Acknowledgments

The authors thank 51runse (www.51runse.cn) for the English language editing during the preparation of this manuscript.

## Author contributions

**Conceptualization:** Cong Chen, Yang Liu.

**Data curation:** Cong Chen.

**Formal analysis:** Songwei Yang.

**Methodology:** Jing Liao, Yang Liu.

**Software:** Ming Chen.

**Visualization:** Ming Chen.

**Writing – original draft:** Cong Chen.

**Writing – review & editing:** Jing Liao.
